# Intention to adhere to test, trace, and isolate during the COVID‐19 pandemic (the COVID‐19 Rapid Survey of Adherence to Interventions and Responses study)

**DOI:** 10.1111/bjhp.12576

**Published:** 2021-11-30

**Authors:** Louise E. Smith, Henry W. W. Potts, Richard Amlȏt, Nicola T. Fear, Susan Michie, G. James Rubin

**Affiliations:** ^1^ Institute of Psychiatry, Psychology and Neuroscience King’s College London UK; ^2^ NIHR Health Protection Research Unit in Emergency Preparedness and Response London UK; ^3^ Institute of Health Informatics University College London UK; ^4^ Behavioural Science and Insights Unit UK Health Security Agency Salisbury UK; ^5^ Academic Department of Military Mental Health King’s Centre for Military Health Research London UK; ^6^ Centre for Behaviour Change University College London UK

**Keywords:** behaviour, contact tracing, COVID‐19, predictors, psychological factors

## Abstract

**Objectives:**

(1) To investigate factors associated with intention to self‐isolate, request a test, and share details of close contacts when required. (2) To determine whether associations were stronger during periods when less stringent national COVID‐19 restrictions were in place.

**Design:**

Series of cross‐sectional nationally representative surveys. We selected survey waves where different national restrictions were in place in England (first lockdown, summer release, second lockdown, third lockdown).

**Methods:**

We investigated whether psychological factors and increased out‐of‐home activity in the last week were associated with intention to self‐isolate and request a test if you were to develop COVID‐19 symptoms, and intention to share details of contacts if you were to test positive. We also investigated whether the strength of associations differed by timepoint in the pandemic.

**Results:**

Intention to self‐isolate, request a test and share details of contacts were associated with greater perceived risk of COVID‐19 to people in the United Kingdom, knowing that COVID‐19 transmission can be asymptomatic, and agreeing that personal behaviour has an impact on COVID‐19 transmission. There were few differences in strength of associations by timepoint suggesting these effects are broadly stable over time.

**Conclusions:**

Psychological factors were associated with intention to adhere to key components of the contact tracing system; there was no evidence for an association with increased out‐of‐home activity. Messages that increase knowledge that COVID‐19 can be transmitted even if someone does not have symptoms and that an individual’s actions can contribute to the spread of the virus may promote engagement with the test, trace, and isolate system.

**Statement of contribution**

***What is already known on this subject?***

- Better engagement with the test, trace, and isolate system in the United Kingdom would reduce transmission and the need for other restrictions.
- Socio‐demographic factors, such as being male and experiencing economic hardship, are associated with non‐adherence to key components of the test, trace, and isolate system, including requesting a test when symptomatic.

***What does this study add?***

- Intention to adhere to key components of the test, trace, and isolate system were associated with greater perceived risk of COVID‐19 to people in the United Kingdom, knowledge that transmission can be asymptomatic, and believing that personal behaviour has an impact on transmission.
- Messages that increase knowledge that COVID‐19 can be spread even if a person does not display symptoms, and that an individual’s actions can contribute to transmission may promote engagement with the test, trace, and isolate system.
- There was no evidence for an association between greater number of outings in the last week (for work or socially) and intention to adhere to test, trace, and isolate if symptomatic.

## Background

In England, people with COVID‐19 symptoms were first asked to self‐isolate on 12 March 2020 (Johnson, 2020a). Everyone with symptoms has been eligible for a test since 18 May 2020 (Department of Health and Social care, 2020), and a contact tracing system, NHS Test and Trace, was launched on 28 May 2020. Despite the test, trace, and isolate system being one of the cornerstones of the UK Government’s response to the COVID‐19 pandemic, engagement with the system has been sub‐optimal. Previous research indicates that at the end of January 2021, approximately 22% of those with COVID‐19 symptoms in the previous week requested a test to see if they had COVID‐19; 52% of those with COVID‐19 symptoms in the previous week and who had not had a negative test result reported fully self‐isolating (Smith et al., 2021). These data do not tell the complete story: qualitative work has suggested that many instances of non‐adherence are relatively low risk with people using the context in which they find themselves to make decisions on how to act. For example, not requesting a test when there is a low probability that a symptom is caused by COVID‐19 (Mowbray, Woodland, Smith, Amlot, & Rubin, 2021) or leaving home during self‐isolation for outdoor exercise but avoiding contact with other people (Denford et al., 2021). Nevertheless, data suggest that many people still report an active intention not to adhere to key elements of Government advice (Smith et al., 2021). When asked to state what actions they would take if they were to develop symptoms of COVID‐19, only 62% reported that they would request a test, 71% reported intending to fully adhere to the rules of self‐isolation, and 79% reported that they would share details of close contacts with NHS Test and Trace if asked to.

Studies investigating factors associated with adherence to test, trace, and isolate have so far focused on investigating associations with socio‐demographic factors, finding that men and people experiencing greater financial hardship are less likely to adhere (Fancourt, Bu, Mak, & Steptoe, 2021; Smith et al., 2021). However, there is limited research investigating the influence of psychological factors despite their likely importance. The Protection Motivation Theory states that appraisal of a threat (perceived susceptibility and severity) and the coping mechanism (perceived effectiveness of the response and one’s ability to carry out that response) influence intention to carry out a health behaviour, which in turn affects actual behaviour (Rogers & Prentice‐Dunn, 1997). Greater perceived risk of COVID‐19 is associated with uptake of protective behaviours (Dryhurst et al., 2020). Knowledge about how COVID‐19 spreads may also affect people’s intention to engage with a contact tracing system. In the context of test, trace, and isolate, knowledge of the symptoms of COVID‐19 among the UK population has previously been shown to be poor (Allington et al., 2020; Smith et al., 2020). Insufficient knowledge about the purpose of quarantine has hindered public health efforts in previous emerging infectious disease outbreaks (Webster et al., 2020). Motivational components to carry out a behaviour may also be influenced by whether information received about the pandemic is viewed as credible (Fancourt, Steptoe, & Wright, 2020; Rubin, Amlot, Page, & Wessely, 2009).

People who have left their home more (for work and to meet others socially) have a greater personal risk of catching COVID‐19, due to increased contact with others. How this may affect intention to engage with a test, trace, and isolate system, if at all, is unclear. People may be more likely to engage, due to greater perceived risk or a normalization of engagement (e.g. routine testing through the workplace), or less likely to engage, due to the possibility that a positive result would stop them from being able to attend work or engage in social activities (Webster et al., 2020).

Complicating our understanding of the factors determining engagement with test, trace, and isolate guidance is the possibility that the relationship between intentions and other psychological factors may change over time. Restrictions in England have changed repeatedly over the course of the pandemic (Box 1) from periods of complete national lockdown to periods in which people were actively incentivized to return to economic activities. Such changes may influence intentions or ‘drown out’ the influence of other variables. For example, adherence to self‐isolation during a period of stringent lockdown may be less a matter of motivation and more a simple reflection of the fact that there are few reasons or opportunities to leave one’s home (Hodson, Woodland, Smith, & Rubin, 2021; Webster et al., 2020).

Box 1 **Timeline of COVID‐19 restrictions in England**

16 March 2020. People asked to stay at home (Johnson, 2020b).

23 March 2020. Lockdown restrictions introduced (could go out only for limited specific reasons; hospitality, non‐essential retail; schools closed) (Johnson, 2020c).

11 May 2020. Restrictions slightly lifted (could go out for exercise as much as want; could mix with one other household outdoors 2 m apart) (Johnson, 2020d).

4 July 2020. Restrictions lifted further (pubs, restaurants re‐opened; could mix with one other household indoors; could stay overnight away from home) (UK Health Security Agency, 2020).

3 to 31 August 2020. Eat Out To Help Out – government subsidies to encourage people to return to hospitality venues (HM Revenue & Customs, 2020).

14 September 2020. Rule of six introduced in indoor and outdoor settings (Home Office, 2020).

14 October 2020. Tier system (1–3) introduced (Scott, 2020).

5 November 2020. Second lockdown restrictions introduced (could go out only for limited specific reasons; hospitality and non‐essential retail closed; schools remained open) (Johnson, 2020e).

2 December 2020. Slightly stricter tier system (1–3) re‐implemented (Hancock, 2020).

19 December 2020. Tier 4 introduced (essentially lockdown restrictions) (Johnson, 2020f).

5 January 2021. Third lockdown restrictions introduced (could go out only for limited specific reasons; hospitality, non‐essential retail and schools closed) (Johnson, 2021).

8 March 2021. Schools re‐opened (Cabinet Office, 2021).

In this study, we investigated whether psychological factors (worry, perceived risk, beliefs about COVID‐19 transmission and personal role, having enough information, perceived credibility of the UK Government) and out‐of‐home activity were associated with intention to engage with the test, trace, and isolate system (intention to self‐isolate, request a test, and share details of close contacts). We also investigated whether the strength of associations differed by timepoint in the pandemic.

## Methods

### Design

BMG Research were conducting a series of nationally representative (UK) cross‐sectional surveys on behalf of the Department of Health and Social Care through the COVID‐19 pandemic. We analysed these data as part of the CORSAIR study (the COVID‐19 Rapid Survey of Adherence to Interventions and Responses study) (Smith et al., 2021). Survey waves were carried out weekly or fortnightly. For this study, we selected waves to capture behaviour during four specific time periods during the pandemic: the first national lockdown (27–29 April 2020 [wave 14] and 4–6 May 2020 [wave 15]), the summer period with fewest restrictions (20–22 July 2020 [wave 25] and 3–5 August 2020 [wave 26]), the second national lockdown (16–18 November 2020 [wave 33] and 23–25 November 2020 [wave 34]), and the third national lockdown (11–13 January 2021 [wave 41] and 25–27 January 2021 [wave 42]).

### Participants

Participants (*n* ≈ 2,000 per wave) were recruited from two specialist research panel providers, Respondi (*n* = 50,000) and Savanta (*n* = 31,500) and were eligible for the study if they were aged 16 years or over and lived in the United Kingdom. Quotas were applied based on age and gender (combined), and reflected targets based on data from the Office for National Statistics (Office for National Statistics, 2019). To avoid the same people taking part in each survey wave, after completing the survey, participants were then unable to participate in the subsequent three waves. Participants were reimbursed in points which could be redeemed in cash, gift vouchers, or charitable donations (up to £0.70 per survey).

For this study, we selected only participants who lived in England due to differing restrictions across the four UK nations. People who reported symptoms in the last week were excluded (first lockdown, *n* = 203; summer, *n* = 211; second lockdown, *n* = 214; third lockdown, *n* = 205) as they were asked about actual, rather than intended behaviour. Therefore, we report on 12,976 responses (first lockdown, *n* = 3,225; summer, *n* = 3,240; second lockdown, *n* = 3,296; third lockdown, *n* = 3,215).

### Study materials

#### Outcome measures

Participants who reported that they had not experienced COVID‐19 symptoms in the last week (high temperature/fever or a new, continuous cough; loss of sense of taste, and loss of sense of smell added on 26 May 2020), were asked to imagine that they developed ‘symptoms of coronavirus’ and asked which actions, if any, they would take. Options included staying at home for 7, 10, or 14 days. From 26 October 2020 (wave 31), these options were replaced with an option to ‘self‐isolate (not leaving the home at all)’. For our self‐isolation outcome, we coded participants as intending to self‐isolate if they selected that they would stay at home for 7, 10, or 14 days, or that they would self‐isolate. Requesting a test to confirm whether you had coronavirus was added to the options of actions on 26 May 2020 (wave 18). For our requesting a test outcome, we coded participants as intending to request a test if they selected the appropriate item.

From 1 June 2020 (wave 19), participants were asked to imagine that they had tested positive for COVID‐19 and been prompted by the NHS contact tracing service and asked how likely they would be to share details of people they had been in close contact with (five‐point scale from ‘definitely would’ to ‘definitely would not’). For our intention to share details of close contacts outcome, we recoded intention into a binary variable, grouping together ‘definitely would’ and ‘probably would’, and ‘not sure’, ‘probably would not’, and ‘definitely would not’.

#### Psychological factors

We asked participants ‘overall, how worried are you about coronavirus’ on a five‐point scale from ‘not at all worried’ to ‘extremely worried’. Participants were asked to what extent they thought COVID‐19 posed a risk to themselves, and to others in the United Kingdom separately, on a five‐point scale from ‘no risk at all’ to ‘major risk’.

To measure beliefs about how COVID‐19 spreads, we asked participants to what extent they agreed that someone could spread coronavirus to other people even if they did not have symptoms yet and that their personal behaviour had an impact on how coronavirus spreads (five‐point scale from ‘strongly disagree’ to ‘strongly agree’).

To investigate having enough information about self‐isolation, testing, and contact tracing programmes, participants were asked to what extent they agreed they had enough information from the Government and other public authorities on a five‐point scale (‘strongly disagree’ to ‘strongly agree’).

We used an adapted form of the Meyer Credibility Index (Cronbach’s α = .83) to measure perceived credibility of information from the Government about COVID‐19 (Meyer, 1988). Participants were asked to what extent they agreed that information from the Government about COVID‐19 could be trusted, was accurate, told the whole story, and was biased or one‐sided.

#### Out‐of‐home activity

We hypothesized that people going out to work were more likely to be in contact with people from other households both at work and on their way to or from work. We asked participants how many times in the last seven days they had been out to meet up with friends or family they did not live with and to go out to work (answers capped at 30 per activity). We summed these values to create a single variable indicating out‐of‐home activity (for work and socially) in the last week.

#### Personal and clinical characteristics

Participants were asked to report their age, gender, employment status, socio‐economic grade, highest educational or professional qualification, ethnicity, marital status, how many people lived in their household, and if there was a dependent child in the household. We also asked participants whether they or a household member had a chronic illness. We coded participants as having a chronic illness that made them clinically vulnerable to COVID‐19 using guidance from the NHS website (NHS, 2020). Participants were asked for their full postcode, from which geographical region and indices of multiple deprivation were determined (Ministry of Housing Communities & Local Government, 2019).

We asked participants if they thought they had previously, or currently, had COVID‐19 on a five‐point scale. We recoded answers into a binary variable: ‘I’ve definitely had it, and had it confirmed by a test’ and ‘ I think I’ve probably had it’, versus ‘I don’t know whether I’ve had it or not’, ‘I think I’ve probably not had it’, and ‘I’ve definitely not had it’.

Financial hardship was measured by asking participants to what extent in the past seven days they had been struggling to make ends meet, skipping meals they would usually have, and were finding their current living situation difficult (Cronbach’s α = .74).

### Ethics

This work was conducted as part of service evaluation of the marketing and communications run by the Department of Health and Social Care, and, following advice from the University Research Ethics Subcommittee, did not require ethical approval.

### Power

A sample size of 3,200 allows a 95% confidence interval of plus or minus 2% for the prevalence estimate for a survey item with a prevalence of around 50%.

### Analysis

We ran χ^2^ analyses to investigate whether outcome variables differed by timepoint in the pandemic.

We used multivariable logistic regression analyses to investigate associations between explanatory variables, socio‐demographic variables, and outcome variables separately for each timepoint in the pandemic (first lockdown; summer; second lockdown; third lockdown). Intention to request a test and share details of close contacts were introduced to the survey after the first lockdown, so we were only able to run these analyses for the latter three timepoints. We created a quadratic term for age, to test for a non‐linear relationship. Multivariable analyses adjusted for survey wave, region (East Midlands arbitrarily allocated as reference category), gender, age (raw and quadratic term), presence of dependent child in the household, being clinically vulnerable to COVID‐19, having a household member with a chronic illness, employment status (working vs. not working), socio‐economic grade (ABC1 vs. C2DE), index of multiple deprivation (quartiles), highest educational or professional qualification (degree or higher vs. less than degree), ethnicity (coded into three categories), living alone, marital status (partnered vs. not partnered), having had COVID‐19 before (think have not had COVID‐19 vs. think or had COVID‐19 confirmed), and financial hardship.

We then conducted further multivariable logistic regression analyses for each outcome variable at each timepoint, entering all factors (personal and clinical characteristics, and explanatory variables) together.

To investigate whether the strength of associations between explanatory variables and outcome variables differed across the pandemic, we conducted meta‐analyses across the timepoints and used an *I* ^2^ statistic to assess heterogeneity. *I* ^2^ estimates the percentage of the variance attributable to heterogeneity, which here means variation across time. Where *I* ^2^ was large (50% or greater), we considered there to be a difference by timepoint in the pandemic; where *I* ^2^ was small (<50%), we determined there was no difference (Patsopoulos, Evangelou, & Ioannidis, 2008).

As multiple analyses were run on individual outcomes (*n* = 7), we applied a Bonferroni correction (*p *< .007).

## Results

Results of fully adjusted models are reported narratively. Analyses controlling only for personal and clinical characteristics are presented in the Appendix S1.

### Intention to self‐isolate

**Figure 1:**
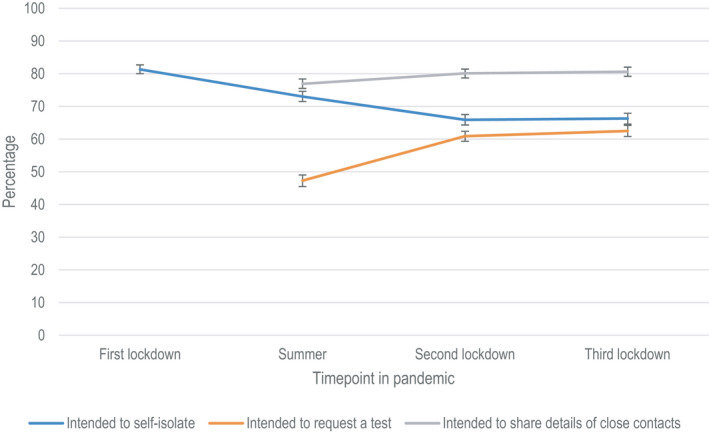
Percentage of people who intended to self‐isolate, request a test, and share details of close contacts at different timepoints in the pandemic.

Intention to self‐isolate differed by timepoint in the pandemic (χ^2^(3) = 251.4, *p *< .001, *n* = 12,976), with intention decreasing over time (Figure 1). In the first lockdown, 81.3% (95% CI 80.0% to 82.7%, *n* = 2,623/3,225) of people intended to self‐isolate. This decreased to 73.0% (95% CI 71.5% to 74.6%, *n* = 2,366/3,240) in the summer, 65.9% (95% CI 64.3%–67.5%, *n* = 2,172/3,296) in the second lockdown, and 66.3% (95% CI 64.6%–67.9%, *n* = 2,130/3,215) in the third lockdown.

**Figure 2:**
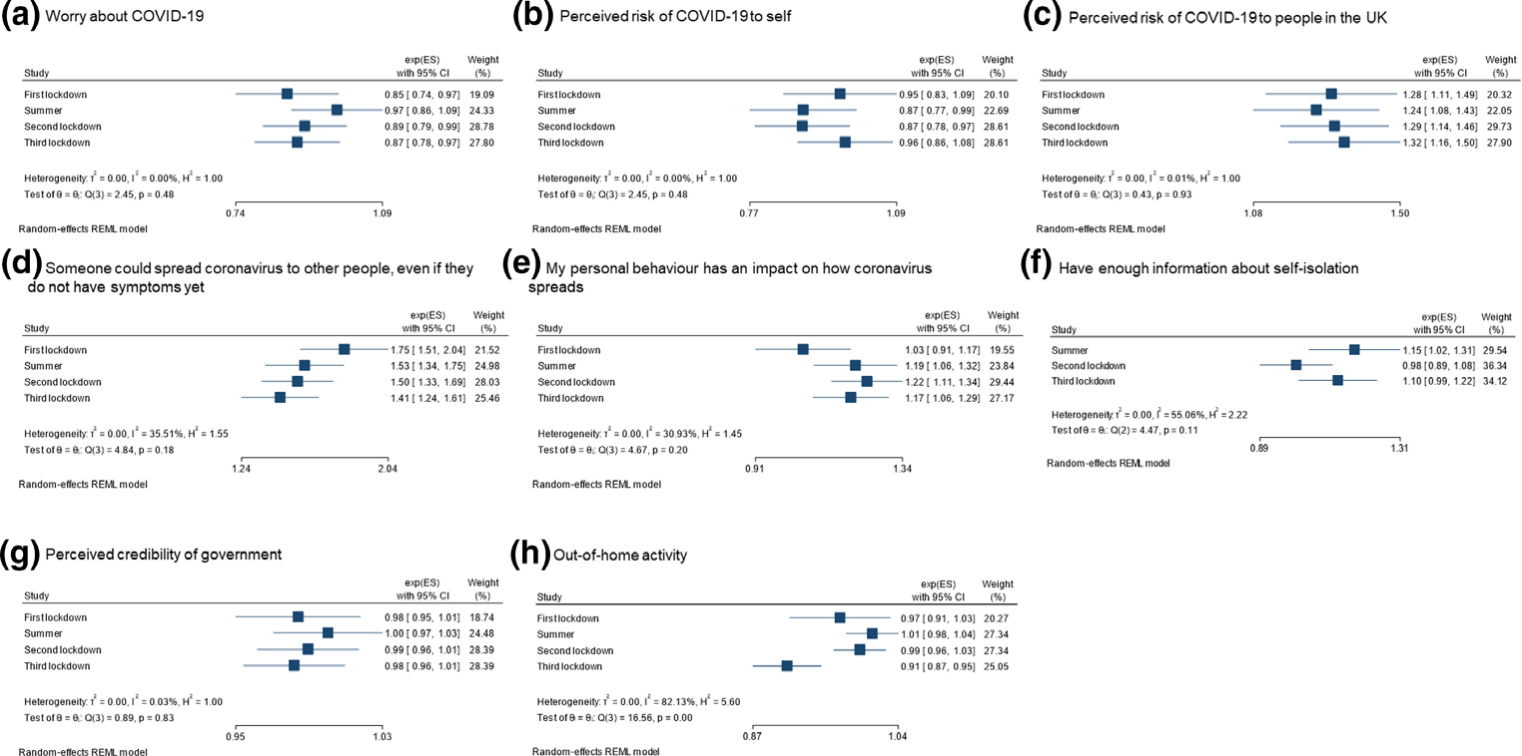
Heterogeneity of strength of associations between psychological factors and intention to self‐isolate at different timepoints in the pandemic.

Figure 2 depicts effect sizes and confidence intervals for associations between explanatory variables and intention to self‐isolate. The *I* ^2^ statistic reflects the percentage of variance in results attributable to heterogeneity, that is to differences over time. There was evidence for substantial heterogeneity in strength of associations between intention to self‐isolate and out‐of‐home activity (*I* ^2^ = 82.1%) and having enough information about self‐isolation during the pandemic (*I* ^2^ = 55.1%). There was minimal evidence for a difference in strength of associations between other explanatory variables and intention to self‐isolate at different timepoints in the pandemic.

In fully adjusted models, intention to self‐isolate was associated with greater perceived risk of COVID‐19 to people in the United Kingdom, knowing that COVID‐19 transmission can be asymptomatic, and agreeing that one's personal behaviour has an impact on the spread of COVID‐19. (Table 1). Going out fewer times for work and socially was also associated with greater intention to self‐isolate in the third lockdown. Personal and clinical characteristics associated with intention to self‐isolate are reported in the Appendix S1.

**Table 1:** Fully adjusted model of factors associated with self‐isolation, for each timepoint in the pandemic

Attribute	Level	First lockdown^a^		Summer, fewest restrictions^b^		Second lockdown^c^		Third lockdown^d^	
		aOR for intending to self‐isolate (95% CI)^e^	*p*	aOR for intending to self‐isolate (95% CI)^f^	*p*	aOR for intending to self‐isolate (95% CI)^f^	*p*	aOR for intending to self‐isolate (95% CI)^f^	*p*
Worry about COVID‐19	5‐point scale (1 = not at all worried to 5 = extremely worried)	0.85 (0.74–0.97)	.02	0.97 (0.86–1.09)	.58	0.89 (0.79–0.99)	.03	0.87 (0.78–0.97)	.02
Perceived risk of COVID‐19 to self	5‐point scale (1 = no risk at all to 5 = major risk)	0.95 (0.83–1.09)	.46	0.87 (0.77–0.99)	.03	0.87 (0.78–0.97)	.01	0.96 (0.86–1.08)	.51
Perceived risk of COVID‐19 to people in the UK	5‐point scale (1 = no risk at all to 5 = major risk)	**1.28 (1.11–1.49)**	.**001**	**1.24 (1.08–1.43)**	.**003**	**1.29 (1.14–1.46)**	**<.001**	**1.32 (1.17–1.50)**	**<.001**
Someone could spread coronavirus to other people, even if they do not have symptoms yet	5‐point scale (1 = strongly disagree to 5 = strongly agree)	**1.75 (1.51–2.04)**	**<.001**	**1.53 (1.34–1.75)**	**<.001**	**1.50 (1.33–1.69)**	**<.001**	**1.41 (1.24–1.61)**	**<.001**
My personal behaviour has an impact on how coronavirus spreads	5‐point scale (1 = strongly disagree to 5 = strongly agree)	1.03 (0.91–1.17)	.59	**1.18 (1.06–1.32)**	.**002**	**1.22 (1.11–1.34)**	**<.001**	**1.17 (1.06–1.29)**	.**001**
Have enough information about self‐isolation	5‐point scale (1 = strongly disagree to 5 = strongly agree)	–	–	1.15 (1.01–1.31)	.03	0.98 (0.89–1.08)	.70	1.10 (0.99–1.22)	.08
Perceived credibility of government	Range 4 (lowest credibility) to 20 (highest credibility)	0.98 (0.95–1.01)	.29	1.00 (0.97–1.03)	.87	0.99 (0.96–1.01)	.36	0.98 (0.96–1.01)	.13
Out‐of‐home activity (for work and socially)	Range 0 (no outings) to 50 (most outings)	0.97 (0.91–1.03)	.32	1.01 (0.98–1.04)	.60	0.99 (0.96–1.03)	.70	**0.91 (0.87–0.95)**	**<.001**

^a^ Model based on 2,694 valid cases (83.5% valid responses).

^b^ Model based on 2,645 valid cases (81.6% valid responses).

^c^ Model based on 2,742 valid cases (83.2% valid responses).

^d^ Model based on 2,704 valid cases (84.1% valid responses).

^e^ All variables entered into regression model together (personal and clinical characteristics, and other psychological factors), excluding perceived adequacy of information about self‐isolation.

^f^ All variables entered into regression model together (personal and clinical characteristics, and other psychological factors), including perceived adequacy of information about self‐isolation.

Bolding indicates findings significant at *p *< 0.007.

### Intention to request a test

Intention to request a test differed by timepoint in the pandemic (χ^2^(2) = 185.6, *p *< .001, *n* = 9,571), with intention increasing over time (Figure 1). In the summer, 47.3% (95% CI 45.5% to 49.0%, *n* = 1,531/3,240) of people intended to request a test if they were to develop symptoms. This increased to 60.9% (95% CI 59.3%–62.4%, *n* = 2,008/3,296) in the second lockdown, and 62.5% (95% CI 60.8%–64.2%, *n* = 2,009/3,215) in the third lockdown.

**Figure 3:**
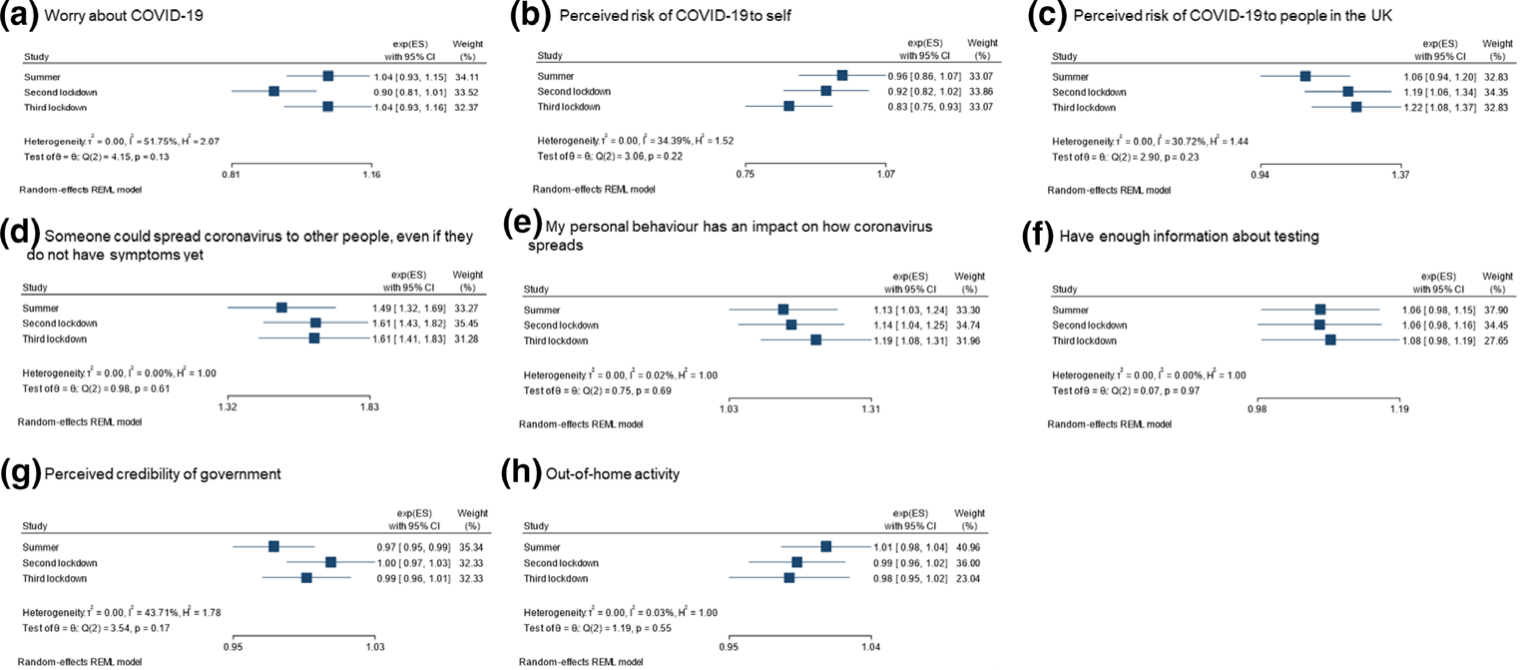
Heterogeneity of strength of associations between psychological factors and intention to request a test at different timepoints in the pandemic.

There was evidence of substantial heterogeneity in strength of associations between intention to request a test and worry about COVID‐19 during the pandemic (*I* ^2^ = 51.8%; Figure 3). There was minimal evidence for a difference in strength of associations between other explanatory variables and intention to request a test at different timepoints in the pandemic.

In fully adjusted models, intention to request a test was associated with greater perceived risk of COVID‐19 to oneself and people in the United Kingdom, knowing that COVID‐19 transmission can be asymptomatic, and agreeing that one's personal behaviour has an impact on the spread of COVID‐19 (Table 2). Personal and clinical characteristics associated with intention to request a test are reported in the Appendix S1.

**Table 2:** Fully adjusted model of factors associated with intention to request a test, for each timepoint in the pandemic

Attribute	Level	Summer, fewest restrictions^a^		Second lockdown^b^		Third lockdown^c^	
		aOR for intending to request a test (95% CI)^d^	*p*	aOR for intending to request a test (95% CI)^d^	*p*	aOR for intending to request a test (95% CI)^d^	*p*
Worry about COVID‐19	5‐point scale (1 = not at all worried to 5 = extremely worried)	1.04 (0.93–1.15)	.49	0.90 (0.81–1.01)	.07	1.04 (0.93–1.16)	.53
Perceived risk of COVID‐19 to self	5‐point scale (1 = no risk at all to 5 = major risk)	0.95 (0.86–1.06)	.40	0.92 (0.82–1.02)	.11	**0.83 (0.75–0.93)**	.**001**
Perceived risk of COVID‐19 to people in the UK	5‐point scale (1 = no risk at all to 5 = major risk)	1.06 (0.94–1.19)	.35	**1.19 (1.06–1.34)**	.**004**	**1.22 (1.08–1.38)**	.**002**
Someone could spread coronavirus to other people, even if they do not have symptoms yet	5‐point scale (1 = strongly disagree to 5 = strongly agree)	**1.49 (1.32–1.69)**	**<.001**	**1.61 (1.43–1.82)**	**<.001**	**1.61 (1.41–1.83)**	**<.001**
My personal behaviour has an impact on how coronavirus spreads	5‐point scale (1 = strongly disagree to 5 = strongly agree)	1.13 (1.02–1.24)	.01	**1.14 (1.04–1.25)**	.**004**	**1.19 (1.08–1.31)**	**<.001**
Have enough information about testing	5‐point scale (1 = strongly disagree to 5 = strongly agree)	1.06 (0.98–1.15)	.13	1.06 (0.98–1.16)	.16	1.08 (0.98–1.19)	.12
Perceived credibility of government	Range 4 (lowest credibility) to 20 (highest credibility)	0.97 (0.95–0.99)	.007	1.00 (0.98–1.02)	.97	0.99 (0.96–1.01)	.27
Out‐of‐home activity (for work and socially)	Range 0 (no outings) to 50 (most outings)	1.01 (0.98–1.04)	.61	0.99 (0.96–1.02)	.50	0.98 (0.95–1.02)	.40

^a^ Model based on 2,646 valid cases (81.7% valid responses).

^b^ Model based on 2,732 valid cases (82.9% valid responses).

^c^ Model based on 2,702 valid cases (84.0% valid responses).

^d^ All variables entered into regression model together (personal and clinical characteristics, and other psychological factors).

Bolding indicates findings significant at *p *< 0.007.

### Intention to share details of close contacts

Intention to share details of close contacts differed by timepoint in the pandemic (χ^2^(2) = 15.3, *p *< .001, *n* = 9,571), with intention slightly increasing over time (Figure 1). In the summer, 76.9% (95% CI 75.5%–78.4%, *n* = 2,493/3,240) of people intended to share details of close contacts if they were to be prompted by the NHS contact tracing service. This increased slightly to 80.1% (95% CI 78.7%–81.4%, *n* = 2,639/3,296) in the second lockdown, and was 80.6% (95% CI 79.2% to 82.0%, *n* = 2,591/3,215) in the third lockdown.

**Figure 4:**
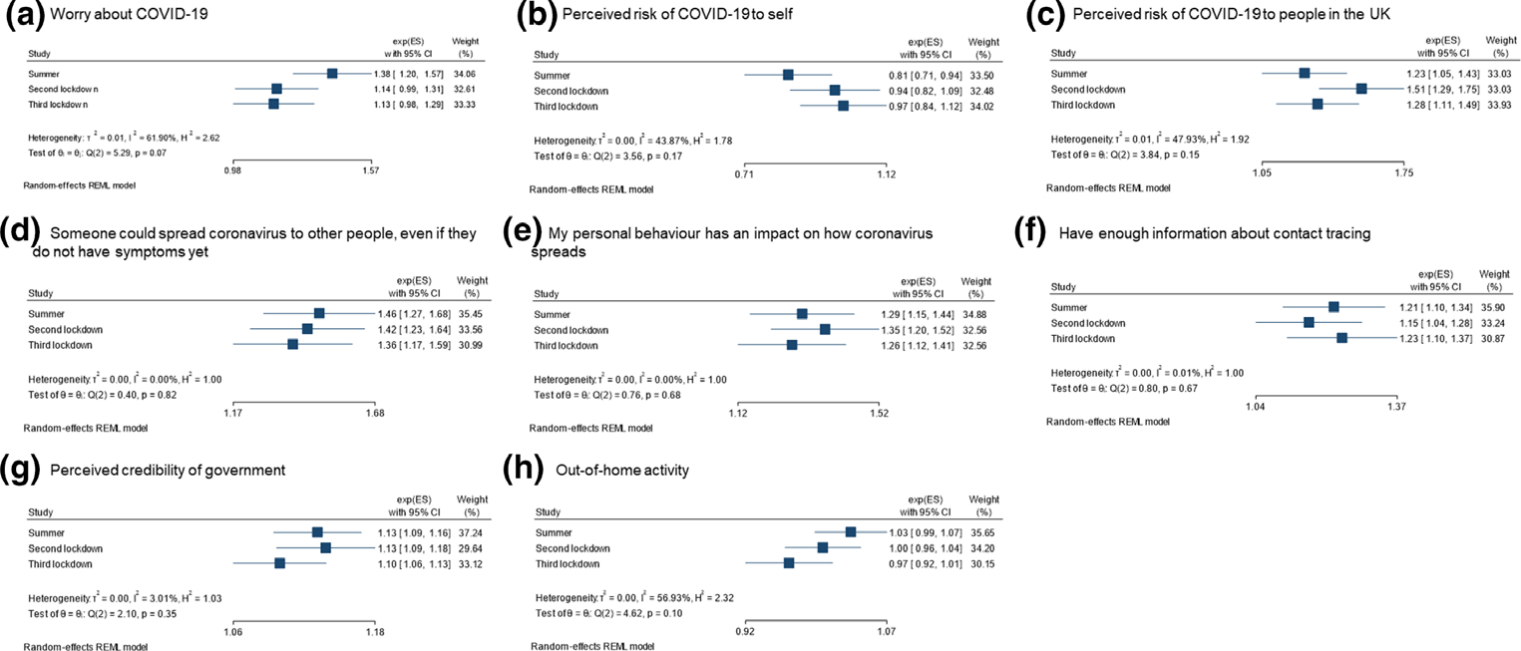
Heterogeneity of strength of associations between psychological factors and intention to share details of close contacts at different timepoints in the pandemic.

There was evidence for substantial heterogeneity in strength of associations between intention to share details of close contacts and worry about COVID‐19 (61.9%), and out‐of‐home activity (*I* ^2^ = 56.9%; Figure 4). There was minimal evidence for a difference in strength of associations between other explanatory variables and intention to share details of close contacts at different timepoints in the pandemic.

In fully adjusted models, intention to share details of close contacts was associated with greater perceived risk of COVID‐19 to oneself and people in the United Kingdom, knowing that COVID‐19 transmission can be asymptomatic, agreeing that one's personal behaviour has an impact on the spread of COVID‐19, agreeing that you had enough information about contact tracing, and greater perceived credibility of the UK Government (Table 3). Greater perceived worry about COVID‐19 was also associated with intending to share details of close contacts in the summer. Personal and clinical characteristics associated with intention to share details of close contacts are reported in the Appendix S1.

**Table 3:** Fully adjusted model of factors associated with intention to share details of close contacts, for each timepoint in the pandemic

Attribute	Level	Summer, fewest restrictions^a^		Second lockdown^b^		Third lockdown^c^	
		aOR for intending to share details (95% CI)^d^	*p*	aOR for intending to share details (95% CI)^d^	*p*	aOR for intending to share details (95% CI)^d^	*p*
Worry about COVID‐19	5‐point scale (1 = not at all worried to 5 = extremely worried)	**1.38 (1.20–1.57)**	**<.001**	1.14 (0.99–1.31)	.07	1.13 (0.98–1.29)	.09
Perceived risk of COVID‐19 to self	5‐point scale (1 = no risk at all to 5 = major risk)	**0.81 (0.70–0.94)**	.**004**	0.94 (0.82–1.09)	.44	0.97 (0.85–1.12)	.68
Perceived risk of COVID‐19 to people in the UK	5‐point scale (1 = no risk at all to 5 = major risk)	1.22 (1.05–1.43)	.009	**1.51 (1.29–1.75)**	**<.001**	**1.28 (1.11–1.49)**	.**001**
Someone could spread coronavirus to other people, even if they do not have symptoms yet	5‐point scale (1 = strongly disagree to 5 = strongly agree)	**1.46 (1.27–1.68)**	**<.001**	**1.42 (1.22–1.64)**	**<.001**	**1.36 (1.17–1.59)**	**<.001**
My personal behaviour has an impact on how coronavirus spreads	5‐point scale (1 = strongly disagree to 5 = strongly agree)	**1.29 (1.15–1.44)**	**<.001**	**1.35 (1.20–1.52)**	**<.001**	**1.26 (1.12–1.41)**	**<.001**
Have enough information about contact tracing	5‐point scale (1 = strongly disagree to 5 = strongly agree)	**1.21 (1.09–1.34)**	**<.001**	1.15 (1.04–1.28)	.008	**1.23 (1.11–1.37)**	**<.001**
Perceived credibility of government	Range 4 (lowest credibility) to 20 (highest credibility)	**1.13 (1.09–1.16)**	**<.001**	**1.13 (1.10–1.17)**	**<.001**	**1.10 (1.06–1.13)**	**<.001**
Out‐of‐home activity (for work and socially)	Range 0 (no outings) to 50 (most outings)	1.03 (0.99–1.07)	.10	1.00 (0.96–1.04)	.91	0.97 (0.92–1.01)	.15

^a^ Model based on 2,640 valid cases (81.5% valid responses).

^b^ Model based on 2,733 valid cases (82.9% valid responses).

^c^ Model based on 2,695 valid cases (83.8% valid responses).

^d^ All variables entered into regression model together (personal and clinical characteristics, and other psychological factors).

Bolding indicates findings significant at *p *< 0.007.

## Discussion

While previous research has investigated associations between socio‐demographic factors and adherence to test, trace, and isolate guidance, there has been little research into associations with potentially modifiable psychological factors. We investigated whether a range of psychological factors and out‐of‐home activity were associated with intention to self‐isolate, request a test, and share details of close contacts when required, and whether associations were stronger during periods when less stringent restrictions were in place.

We found few differences in strength of associations by timepoint in the pandemic. While some factors showed evidence of heterogeneity in the strength of associations with outcomes over the pandemic, in practice there was little evidence for associations between outcomes and these factors. The only exceptions were for worry (which was associated with intention to share details of contacts in the summer, but not in the second or third lockdown) and out‐of‐home activity (which was associated with lower intention to self‐isolate in the third lockdown, but not in any other period). Even in these instances, however, the odds ratios that we found for each period were very similar, suggesting effects are broadly stable over time. This is in line with other research investigating predictors of health behaviours in the United Kingdom during the pandemic (Schneider et al., 2021), indicating that results from the start of the pandemic are still valid to inform communications in later stages.

Intention to self‐isolate, request a test, and share details of close contacts were all associated with greater perceived risk to people in the United Kingdom, but not oneself. Given that test, trace, and isolate is intended to protect other people when a person is infected with COVID‐19, this pattern of results makes sense. It is notable that perceived risk to others, and not to oneself, has also been reported as a motivation for vaccination in the United Kingdom (Sherman et al., 2021). Results suggest that a desire to protect others may be a more fundamental driver of behaviour during the COVID‐19 pandemic. Messaging promoting that completing protective behaviours will keep others safe may promote adherence.

Knowing that COVID‐19 transmission can be asymptomatic was also associated with intending to self‐isolate, request a test, and share details of close contacts. This is in line with theoretical models of the uptake of health behaviour, such as the COM‐B model, which posit a role for knowledge (through psychological capability) in determining behaviour (Michie, van Stralen, & West, 2011). Greater knowledge about transmission and treatment was also associated with uptake of protective behaviours (maintaining distance, wearing a face covering, and hand washing) in another study (Rattay et al., 2021). For our data, we speculate that greater understanding of the risk of asymptomatic transmission could be associated with a greater belief that COVID‐19 is easy to transmit, making test, trace, and isolate appear more important.

Agreeing that your personal behaviour has an impact on the spread of COVID‐19 was associated with intending to self‐isolate, request a test, and share details of close contacts. Internal locus of control is associated with health behaviours more generally (Norman, Bennett, Smith, & Murphy, 1998). Although there is little research investigating locus of control with respect to COVID‐19, at least one study has suggested that internal locus of control is associated with intending to engage in various behaviours including handwashing, social distancing, wearing a face covering, and staying at home apart from essential reasons (Devereux, Miller, & Kirshenbaum, 2021). Perceived behavioural control was also the strongest predictor of high uptake of preventive behaviours in a separate study after adjusting for socio‐demographic characteristics, perceiving risk, attitudes towards the behaviour, and subjective norms (Mao et al., 2021). Potentially, focussing on someone’s agency in preventing the spread of infection may be a useful strategy for communications around test, trace, and isolate (Porat, Nyrup, Calvo, Paudyal, & Ford, 2020).

Limitations of this study include that we measured intention, rather than actual behaviour. The intention‐behaviour gap posits that rates of people carrying out a behaviour are likely to be lower than the rate intending to carry it out (Smith et al., 2021; Sniehotta, Scholz, & Schwarzer, 2005). However, our finding that a sizeable minority of respondents report that they do not intend to engage with test, trace, or isolate behaviours is an important finding in its own right. Quota sampling was used to generate a sample whose socio‐demographic characteristics were broadly representative of the United Kingdom population. While we cannot be certain that the views and intended behaviours of people who complete online surveys are representative of the general population, we assume that associations between variables follow the same pattern as in the general population (Kohler, 2019). Perceived risk to self could have interacted with vaccination status, but we did not include vaccination status as a variable in analyses. As the COVID‐19 vaccination programme was only initiated in England in December 2020, this would only have affected data collected during the third national lockdown. At the time of our data collection, only 6,473,752 first doses and 445,101 second doses of the vaccine had been delivered in England [total population 56 million] (GOV.UK, 2021). Priority groups for vaccination in the United Kingdom at that time were people aged 80 years and older, residents in care homes, and health and social care staff (Department of Health & Social Care, 2021; Joint Committee on Vaccination and Immunisation, 2020). We did not measure all factors that could theoretically have been associated with intention, such as perceived effectiveness and self‐efficacy for behaviours. This was due to space limitations in the questionnaire. These could have influenced our results. For example, a service evaluation of NHS Test and Trace in Wales found that adherence to self‐isolation was greater in those who had higher confidence in their ability to self‐isolate (Kyle, Isherwood, Bailey, & Davies, 2021). In this study, we did not assess participants’ knowledge of the guidance on test, trace, and isolate. If people are unaware of the guidance, it follows that they may be unlikely to intend to follow it. Analyses relating to knowledge of self‐isolation requirements are reported separately (Smith et al., 2021).

Intention to adhere to key components of the test, trace, and isolate system was associated with psychological factors. Using a theoretical framework, these factors were relevant to reflective motivation and psychological capability. There were few differences in strength of associations by timepoint in the pandemic. Intentions to self‐isolate, request a test, and share details of contacts were associated with greater perceived risk of COVID‐19 to people in the United Kingdom, knowing that COVID‐19 transmission can be asymptomatic, and agreeing that one's personal behaviour has an impact on COVID‐19 transmission. Communications should aim to increase knowledge that COVID‐19 can be transmitted even if someone does not have symptoms, promote perceived control over transmission, and highlight that adhering to protective behaviours will protect others; these may encourage adoption of preventive behaviours.

## Funding information

All authors had financial support from NIHR for the submitted work. RA is an employee of the UK Health Security Agency; HWWP has received additional salary support from Public Health England and NHS England; HWWP receives consultancy fees to his employer from Ipsos MORI and has a PhD student who works at and has fees paid by AstraZeneca; NTF is a participant of an independent group advising NHS Digital on the release of patient data. All authors are participants of the UK’s Scientific Advisory Group for Emergencies or its subgroups. There are no other financial relationships with any organizations that might have an interest in the submitted work in the previous three years and no other relationships or activities that could appear to have influenced the submitted work.

## Declaration of competing interests

This work was funded by the National Institute for Health Research (NIHR) Health Services and Delivery Research programme. LS, RA, and GJR are supported by the National Institute for Health Research Health Protection Research Unit (NIHR HPRU) in Emergency Preparedness and Response, a partnership between the UK Health Security Agency, King’s College London and the University of East Anglia. RA is also supported by the NIHR HPRU in Behavioural Science and Evaluation, a partnership between the UK Health Security Agency and the University of Bristol. HWWP has received funding from Public Health England and NHS England. NTF is part funded by a grant from the UK Ministry of Defence. The views expressed are those of the authors and not necessarily those of the NIHR, the UK Health Security Agency, the Department of Health and Social Care or the Ministry of Defence. Surveys were commissioned and funded by Department of Health and Social Care (DHSC), with the authors providing advice on the question design and selection. Preliminary results were made available to DHSC and the UK’s Scientific Advisory Group for Emergencies.

## Author contributions

**Louise E Smith:** Conceptualization (equal); Data curation (equal); Formal analysis (equal); Methodology (equal); Writing – original draft (equal). **Henry WW Potts:** Conceptualization (equal); Formal analysis (equal); Funding acquisition (equal); Methodology (equal); Writing – review & editing (equal). **Richard Amlot:** Conceptualization (equal); Funding acquisition (equal); Methodology (equal); Writing – review & editing (equal). **Nicola T Fear:** Conceptualization (equal); Funding acquisition (equal); Methodology (equal); Writing – review & editing (equal). **Susan Michie:** Conceptualization (equal); Funding acquisition (equal); Methodology (equal); Writing – review & editing (equal). **G James Rubin:** Conceptualization (equal); Funding acquisition (equal); Methodology (equal); Writing – review & editing (equal).

## Data availability statement

The data are owned by the UK’s Department of Health and Social Care, so no additional data are available from the authors.

## Supporting information


**Appendix S1.** Supplementary results.
