# Supplementary material for: Intention to adhere to test, trace, and isolate during the COVID‐19 pandemic (the COVID‐19 Rapid Survey of Adherence to Interventions and Responses study)
Source: Br J Health Psychol. 2021 Nov 30;27(3):1100–18. doi: 10.1111/bjhp.12576 (PMC9542361; doi:10.1111/bjhp.12576)
Supplement: Supplementary file 1 — Appendix S1. Supplementary results. [file BJHP-27-1100-s001.docx]

# Supplementary materials. Results of regression analyses controlling only for personal and clinical characteristics.

## Intention to self-isolate

Table 1. Factors associated with intention to self-isolate, adjusting for personal and clinical characteristics, for each timepoint in the pandemic. Bolding indicates findings significant at p<0.007.

|  |  | First lockdown | | | | Summer, fewest restrictions | | | | Second lockdown | | | | Third lockdown | | | |
| --- | --- | --- | --- | --- | --- | --- | --- | --- | --- | --- | --- | --- | --- | --- | --- | --- | --- |
| Attribute | Level | Did not intend to self-isolate n=602 | Intended to self-isolate n=2623 | aOR for intending to self-isolate (95% CI) † | *p* | Did not intend to self-isolate n=874 | Intended to self-isolate n=2366 | aOR for intending to self-isolate (95% CI) † | *p* | Did not intend to self-isolate n=1124 | Intended to self-isolate n=2172 | aOR for intending to self-isolate (95% CI) † | *p* | Did not intend to self-isolate n=1085 | Intended to self-isolate n=2130 | aOR for intending to self-isolate (95% CI) † | *p* |
| Worry about COVID-19 | 5-point scale (1=not at all worried to 5=extremely worried) | N=598, M=3.6, SD=1.2 | N=2619, M=3.6, SD=1.0 | 1.01 (0.92 to 1.12) | .79 | N=863, M=3.3, SD=1.2 | N=2358, M=3.4, SD=1.0 | **1.13 (1.05 to 1.23)** | **.002** | N=1116, M=3.3, SD=1.2 | N=2167, M=3.5, SD=1.0 | 1.07 (1.00 to 1.16) | .06 | N=1080, M=3.6, SD=1.2 | N=2129, M=3.7, SD=1.1 | 1.10 (1.02 to 1.19) | .01 |
| Perceived risk of COVID-19 to self | 5-point scale (1=no risk at all to 5=major risk) | N=588, M=3.2, SD=1.2 | N=2599, M=3.2, SD=1.1 | 1.06 (0.97 to 1.17) | .21 | N=848, M=3.0, SD=1.2 | N=2343, M=3.1, SD=1.1 | 1.09 (1.01 to 1.19) | .03 | N=1087, M=3.1, SD=1.2 | N=2155, M=3.2, SD=1.1 | 1.05 (0.97 to 1.13) | .24 | N=1063, M=3.2, SD=1.2 | N=2115, M=3.4, SD=1.1 | **1.12 (1.04 to 1.21)** | **.004** |
| Perceived risk of COVID-19 to people in the UK | 5-point scale (1=no risk at all to 5=major risk) | N=586, M=3.7, SD=1.0 | N=2605, M=3.9, SD=0.9 | **1.24 (1.11 to 1.38)** | **<.001** | N=846, M=3.4, SD=1.1 | N=2349, M=3.7, SD=0.9 | **1.32 (1.19 to 1.45)** | **<.001** | N=1095, M=3.6, SD=1.0 | N=2154, M=3.8, SD=0.9 | **1.26 (1.16 to 1.37)** | **<.001** | N=1065, M=3.8, SD=1.0 | N=2122, M=4.1, SD=0.9 | **1.38 (1.26 to 1.50)** | **<.001** |
| Someone could spread coronavirus to other people, even if they do not have symptoms yet | 5-point scale (1=strongly disagree to 5=strongly agree) | N=584, M=4.1, SD=0.9 | N=2589, M=4.6, SD=0.7 | **1.79 (1.57 to 2.03)** | **<.001** | N=851, M=4.0, SD=0.9 | N=2346, M=4.4, SD=0.7 | **1.71 (1.53 to 1.92)** | **<.001** | N=1109, M=4.1, SD=0.9 | N=2157, M=4.4, SD=0.7 | **1.62 (1.46 to 1.80)** | **<.001** | N=1070, M=4.2, SD=0.8 | N=2115, M=4.5, SD=0.7 | **1.67 (1.49 to 1.86)** | **<.001** |
| My personal behaviour has an impact on how coronavirus spreads | 5-point scale (1=strongly disagree to 5=strongly agree) | N=582, M=3.9, SD=1.1 | N=2590, M=4.2, SD=1.0 | **1.25 (1.14 to 1.38)** | **<.001** | N=852, M=3.7, SD=1.0 | N=2342, M=4.1, SD=0.9 | **1.39 (1.28 to 1.52)** | **<.001** | N=1108, M=3.7, SD=1.0 | N=2153, M=4.1, SD=1.0 | **1.37 (1.26 to 1.48)** | **<.001** | N=1072, M=3.9, SD=1.0 | N=2120, M=4.2, SD=1.0 | **1.32 (1.22 to 1.43)** | **<.001** |
| Have enough information about self-isolation | 5-point scale (1=strongly disagree to 5=strongly agree) | - | - | - | - | N=852, M=3.9, SD=0.9 | N=2348, M=4.1, SD=0.8 | **1.37 (1.23 to 1.52)** | **<.001** | N=1108, M=3.8, SD=1.0 | N=2160, M=4.0, SD=0.9 | 1.10 (1.01 to 1.20) | .03 | N=1064, M=3.9, SD=0.9 | N=2121, M=4.1, SD=0.9 | **1.23 (1.12 to 1.34)** | **<.001** |
| Perceived credibility of government | Range 4 (lowest credibility) to 20 (highest credibility) | N=565, M=13.0, SD=3.4 | N=2445, M=13.4, SD=3.6 | 1.00 (0.97 to 1.03) | .94 | N=811, M=11.7, SD=3.6 | N=2221, M=11.9, SD=3.8 | 1.01 (0.99 to 1.04) | .23 | N=1052, M=11.4, SD=3.6 | N=2054, M=11.6, SD=3.8 | 1.01 (0.99 to 1.03) | .53 | N=1010, M=12.1, SD=3.7 | N=2027, M=12.4, SD=3.9 | 1.01 (0.99 to 1.04) | .27 |
| Out-of-home activity (for work and socially) | Range 0 (no outings) to 50 (most outings) | N=602, M=0.9, SD=2.0 | N=2623, M=0.6, SD=1.7 | 0.97 (0.92 to 1.03) | .33 | N=874, M=2.4, SD=3.4 | N=2366, M=1.9, SD=2.7 | 1.00 (0.97 to 1.03) | .89 | N=1124, M=2.0, SD=3.2 | M=2172, M=1.5, SD=2.7 | 0.99 (0.96 to 1.02) | .37 | N=1085, M=1.6, SD=3.0 | N=2130, M=1.0, SD=2.0 | **0.91 (0.88 to 0.95)** | **<.001** |

† Adjusting for survey wave, region, gender, age (raw and quadratic term), dependent child in the household, being clinically vulnerable to COVID-19, having a household member with a chronic illness, employment status, socio-economic grade, index of multiple deprivation, highest educational or professional qualification, ethnicity, living alone, marital status, ever had COVID-19 and financial hardship.

## Intention to request a test

Table 2. Factors associated with intention to request a test, adjusting for personal and clinical characteristics, for each timepoint in the pandemic. Bolding indicates findings significant at p<0.007.

|  |  | Summer, fewest restrictions | | | | Second lockdown | | | | Third lockdown | | | |
| --- | --- | --- | --- | --- | --- | --- | --- | --- | --- | --- | --- | --- | --- |
| Attribute | Level | Did not intend to request a test n=1709 | Intended to request a test n=1531 | aOR for intending to request a test (95% CI) † | *p* | Did not intend to request a test n=1288 | Intended to request a test n=2008 | aOR for intending to request a test (95% CI) † | *p* | Did not intend to request a test n=1206 | Intended to request a test n=2009 | aOR for intending to request a test (95% CI) † | *p* |
| Worry about COVID-19 | 5-point scale (1=not at all worried to 5=extremely worried) | N=1694, M=3.3, SD=1.2 | N=1527, M=3.4, SD=1.0 | **1.13 (1.05 to 1.21)** | **.001** | N=1278, M=3.4, SD=1.2 | N=2005, M=3.5, SD=1.0 | 1.09 (1.02 to 1.17) | .02 | N=1202, M=3.5, SD=1.2 | N=2007, M=3.7, SD=1.0 | **1.21 (1.12 to 1.30)** | **<.001** |
| Perceived risk of COVID-19 to self | 5-point scale (1=no risk at all to 5=major risk) | N=1675, M=3.0, SD=1.2 | N=1516, M=3.1, SD=1.0 | **1.11 (1.03 to 1.20)** | **.005** | N=1246, M=3.1, SD=1.2 | N=1996, M=3.2, SD=1.1 | 1.06 (0.98 to 1.14) | .13 | N=1181, M=3.3, SD=1.2 | N=1997, M=3.4, SD=1.1 | 1.09 (1.01 to 1.17) | .03 |
| Perceived risk of COVID-19 to people in the UK | 5-point scale (1=no risk at all to 5=major risk) | N=1670, M=3.6, SD=1.0 | N=1525, M=3.7, SD=0.9 | **1.18 (1.08 to 1.28)** | **<.001** | N=1254, M=3.6, SD=1.1 | N=1995, M=3.8, SD=0.9 | **1.22 (1.12 to 1.32)** | **<.001** | N=1189, M=3.9, SD=1.0 | N=1998, M=4.1, SD=0.8 | **1.32 (1.21 to 1.44)** | **<.001** |
| Someone could spread coronavirus to other people, even if they do not have symptoms yet | 5-point scale (1=strongly disagree to 5=strongly agree) | N=1681, M=4.2, SD=0.9 | N=1516, M=4.5, SD=0.6 | **1.59 (1.42 to 1.77)** | **<.001** | N=1268, M=4.1, SD=0.9 | N=1998, M=4.5, SD=0.7 | **1.73 (1.56 to 1.92)** | **<.001** | N=1192, M=4.2, SD=0.9 | N=1993, M=4.6, SD=0.6 | **1.82 (1.62 to 2.03)** | **<.001** |
| My personal behaviour has an impact on how coronavirus spreads | 5-point scale (1=strongly disagree to 5=strongly agree) | N=1676, M=3.9, SD=1.0 | N=1518, M=4.1, SD=0.9 | **1.26 (1.16 to 1.36)** | **<.001** | N=1269, M=3.8, SD=1.1 | N=1992, M=4.1, SD=1.0 | **1.30 (1.21 to 1.41)** | **<.001** | N=1193, M=3.9, SD=1.0 | N=1999, M=4.3, SD=0.9 | **1.34 (1.24 to 1.46)** | **<.001** |
| Have enough information about testing | 5-point scale (1=strongly disagree to 5=strongly agree) | N=1681, M=3.5, SD=1.1 | N=1519, M=3.5, SD=1.1 | 1.06 (0.99 to 1.14) | .09 | N=1262, M=3.4, SD=1.1 | N=1986, M=3.5, SD=1.1 | **1.11 (1.03 to 1.19)** | **.005** | N=1172, M=3.6, SD=1.0 | N=1998, M=3.8, SD=1.0 | **1.14 (1.05 to 1.24)** | **.001** |
| Perceived credibility of government | Range 4 (lowest credibility) to 20 (highest credibility) | N=1584, M=12.0, SD=3.7 | N=1448, M=11.7, SD=3.8 | 0.98 (0.96 to 1.00) | .12 | N=1211, M=11.3, SD=3.8 | N=1895, M=11.7, SD=3.7 | 1.02 (1.00 to 1.04) | .08 | N=1129, M=12.0, SD=3.8 | N=1908, M=12.5, SD=3.8 | 1.02 (1.00 to 1.04) | .04 |
| Out-of-home activity (for work and socially) | Range 0 (no outings) to 50 (most outings) | N=1709, M=2.2, SD=3.2 | N=1531, M=2.0, SD=2.6 | 1.00 (0.98 to 1.03) | .77 | N=1288, M=1.8, SD=3.5 | N=2008, M=1.6, SD=2.5 | 0.98 (0.95 to 1.01) | .16 | N=1206, M=1.3, SD=2.7 | M=2009, M=1.2, SD=2.2 | 0.97 (0.94 to 1.01) | .15 |

† Adjusting for survey wave, region, gender, age (raw and quadratic term), dependent child in the household, being clinically vulnerable to COVID-19, having a household member with a chronic illness, employment status, socio-economic grade, index of multiple deprivation, highest educational or professional qualification, ethnicity, living alone, marital status, ever had COVID-19 and financial hardship.

## Intention to share details of close contacts

Table 3. Factors associated with intention to share details of close contacts, adjusting for personal and clinical characteristics, for each timepoint in the pandemic. Bolding indicates findings significant at p<0.007.

|  |  | Summer, fewest restrictions | | | | Second lockdown | | | | Third lockdown | | | |
| --- | --- | --- | --- | --- | --- | --- | --- | --- | --- | --- | --- | --- | --- |
| Attribute | Level | Did not intend to share details n=747 | Intended to share details n=2493 | aOR for intending to share details (95% CI) † | *p* | Did not intend to share details n=657 | Intended to share details n=2639 | aOR for intending to share details (95% CI) † | *p* | Did not intend to share details n=624 | Intended to share details n=2591 | aOR for intending to share details (95% CI) † | *p* |
| Worry about COVID-19 | 5-point scale (1=not at all worried to 5=extremely worried) | N=737, M=3.0, SD=1.2 | N=2484, M=3.5, SD=1.0 | **1.50 (1.38 to 1.64)** | **<.001** | N=646, M=2.9, SD=1.2 | N=2637, M=3.5, SD=1.0 | **1.75 (1.60 to 1.92)** | **<.001** | N=620, M=3.2, SD=1.3 | N=2589, M=3.8, SD=1.0 | **1.56 (1.43 to 1.71)** | **<.001** |
| Perceived risk of COVID-19 to self | 5-point scale (1=no risk at all to 5=major risk) | N=722, M=2.8, SD=1.2 | N=2469, M=3.2, SD=1.1 | **1.26 (1.15 to 1.38)** | **<.001** | N=628, M=2.8, SD=1.1 | N=2614, M=3.3, SD=1.1 | **1.60 (1.45 to 1.77)** | **<.001** | N=609, M=3.0, SD=1.2 | N=2569, M=3.4, SD=1.1 | **1.44 (1.31 to 1.58)** | **<.001** |
| Perceived risk of COVID-19 to people in the UK | 5-point scale (1=no risk at all to 5=major risk) | N=717, M=3.3, SD=1.0 | N=2478, M=3.7, SD=0.9 | **1.50 (1.36 to 1.66)** | **<.001** | N=633, M=3.3, SD=1.1 | N=2616, M=3.8, SD=0.9 | **2.01 (1.81 to 2.23)** | **<.001** | N=608, M=3.6, SD=1.1 | N=2579, M=4.1, SD=0.8 | **1.75 (1.58 to 1.95)** | **<.001** |
| Someone could spread coronavirus to other people, even if they do not have symptoms yet | 5-point scale (1=strongly disagree to 5=strongly agree) | N=722, M=4.0, SD=0.9 | N=2475, M=4.4, SD=0.7 | **1.80 (1.60 to 2.02)** | **<.001** | N=643, M=3.9, SD=0.9 | N=2623, M=4.4, SD=0.7 | **1.90 (1.69 to 2.13)** | **<.001** | N=612, M=4.0, SD=1.0 | N=2573, M=4.5, SD=0.7 | **1.93 (1.71 to 2.19)** | **<.001** |
| My personal behaviour has an impact on how coronavirus spreads | 5-point scale (1=strongly disagree to 5=strongly agree) | N=724, M=3.6, SD=1.1 | N=2470, M=4.1, SD=0.9 | **1.59 (1.45 to 1.74)** | **<.001** | N=635, M=3.5, SD=1.0 | N=2626, M=4.1, SD=1.0 | **1.73 (1.58 to 1.89)** | **<.001** | N=613, M=3.7, SD=1.1 | N=2579, M=4.2, SD=0.9 | **1.62 (1.48 to 1.78)** | **<.001** |
| Have enough information about contact tracing | 5-point scale (1=strongly disagree to 5=strongly agree) | N=723, M=3.1, SD=1.1 | N=2464, M=3.5, SD=1.1 | **1.41 (1.30 to 1.53)** | **<.001** | N=640, M=3.0, SD=1.2 | N=2605, M=3.5, SD=1.1 | **1.44 (1.32 to 1.56)** | **<.001** | N=603, M=3.0, SD=1.1 | N=2567, M=3.5, SD=1.1 | **1.47 (1.35 to 1.60)** | **<.001** |
| Perceived credibility of government | Range 4 (lowest credibility) to 20 (highest credibility) | N=690, M=10.5, SD=3.5 | N=2342, M=12.2, SD=3.7 | **1.15 (1.12 to 1.18)** | **<.001** | N=605, M=9.7, SD=3.6 | N=2501, M=12.0, SD=3.6 | **1.19 (1.16 to 1.22)** | **<.001** | N=577, M=10.5, SD=3.8 | N=2460, M=12.7, SD=3.7 | **1.17 (1.13 to 1.20)** | **<.001** |
| Out-of-home activity (for work and socially) | Range 0 (no outings) to 50 (most outings) | N=747, M=2.3, SD=3.3 | N=2493, M=2.0, SD=2.8 | 1.01 (0.98 to 1.05) | .38 | M=657, M=2.0, SD=2.9 | N=2639, M=1.6, SD=2.9 | 0.98 (0.95 to 1.01) | .16 | N=624, M=1.5, SD=3.1 | N=2591, M=1.2, SD=2.2 | 0.95 (0.92 to 0.99) | .02 |

† Adjusting for survey wave, region, gender, age (raw and quadratic term), dependent child in the household, being clinically vulnerable to COVID-19, having a household member with a chronic illness, employment status, socio-economic grade, index of multiple deprivation, highest educational or professional qualification, ethnicity, living alone, marital status, ever had COVID-19 and financial hardship.

# Supplementary materials. Results of heterogeneity analyses controlling only for personal and clinical characteristics.

## Intention to self-isolate

Figure 1. Heterogeneity of strength of associations between psychological factors and intention to self-isolate at different timepoints in the pandemic, when controlling for personal and clinical characteristics.


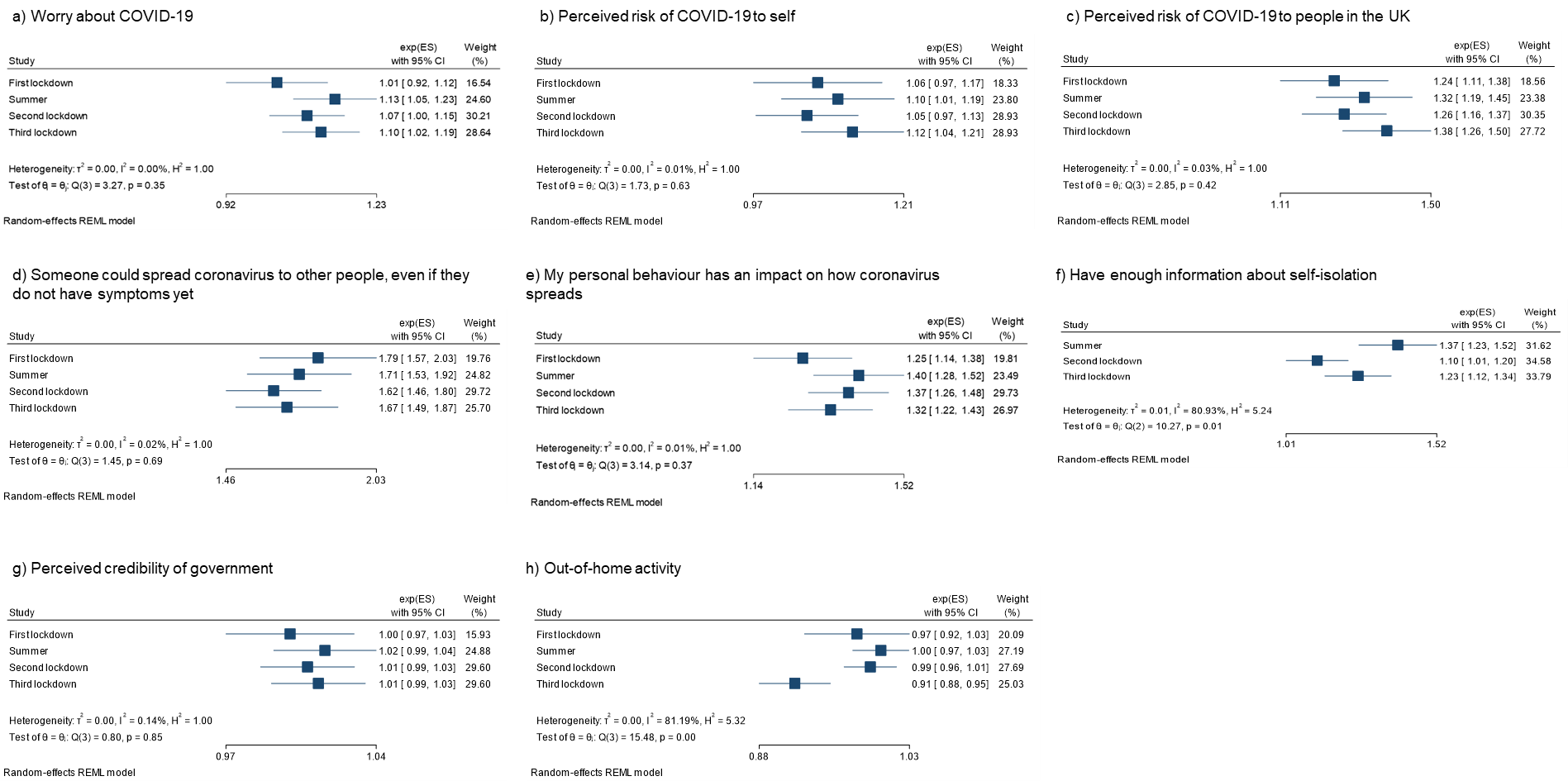


## Intention to request a test

Figure 2. Heterogeneity of strength of associations between psychological factors and intention to request a test at different timepoints in the pandemic, when controlling for personal and clinical characteristics.


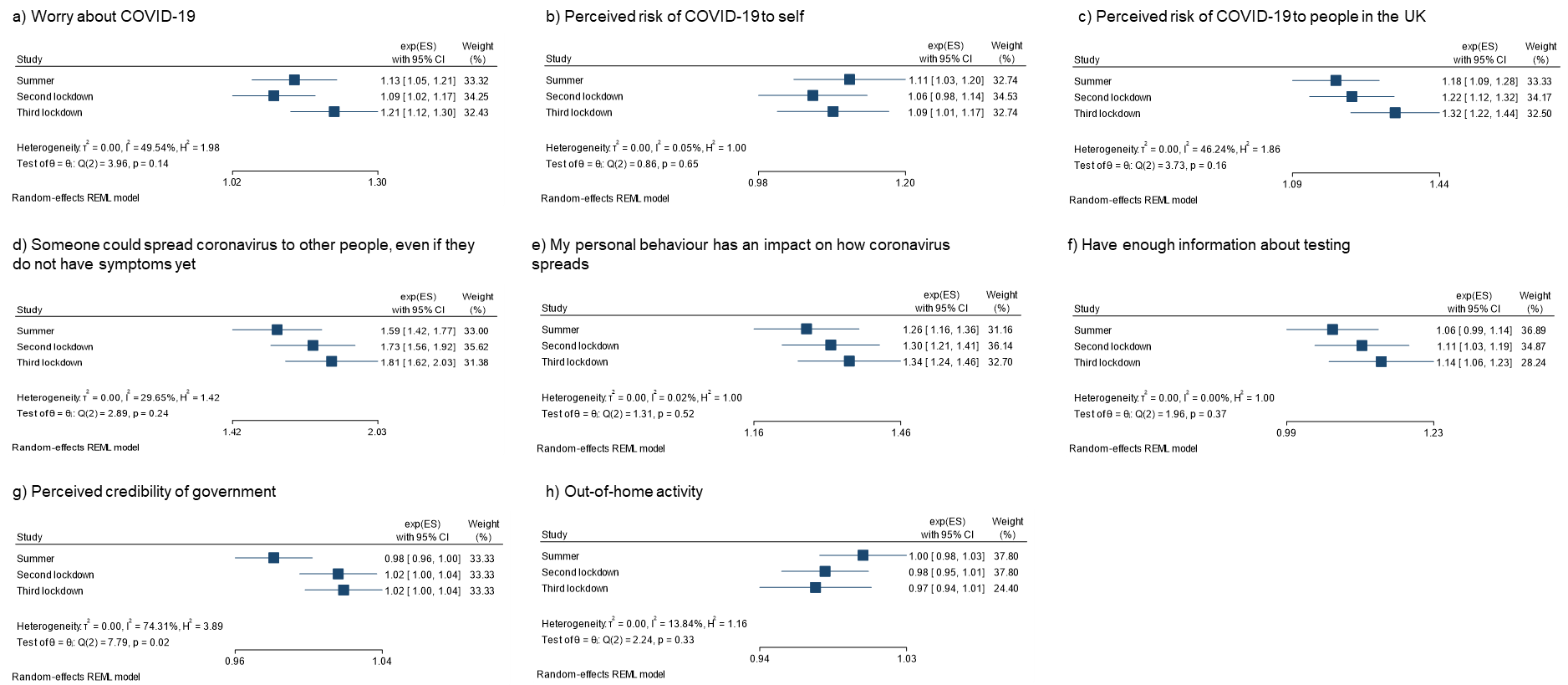


## Intention to share details of close contacts

Figure 3. Heterogeneity of strength of associations between psychological factors and intention to share details of close contacts at different timepoints in the pandemic, when controlling for personal and clinical characteristics.


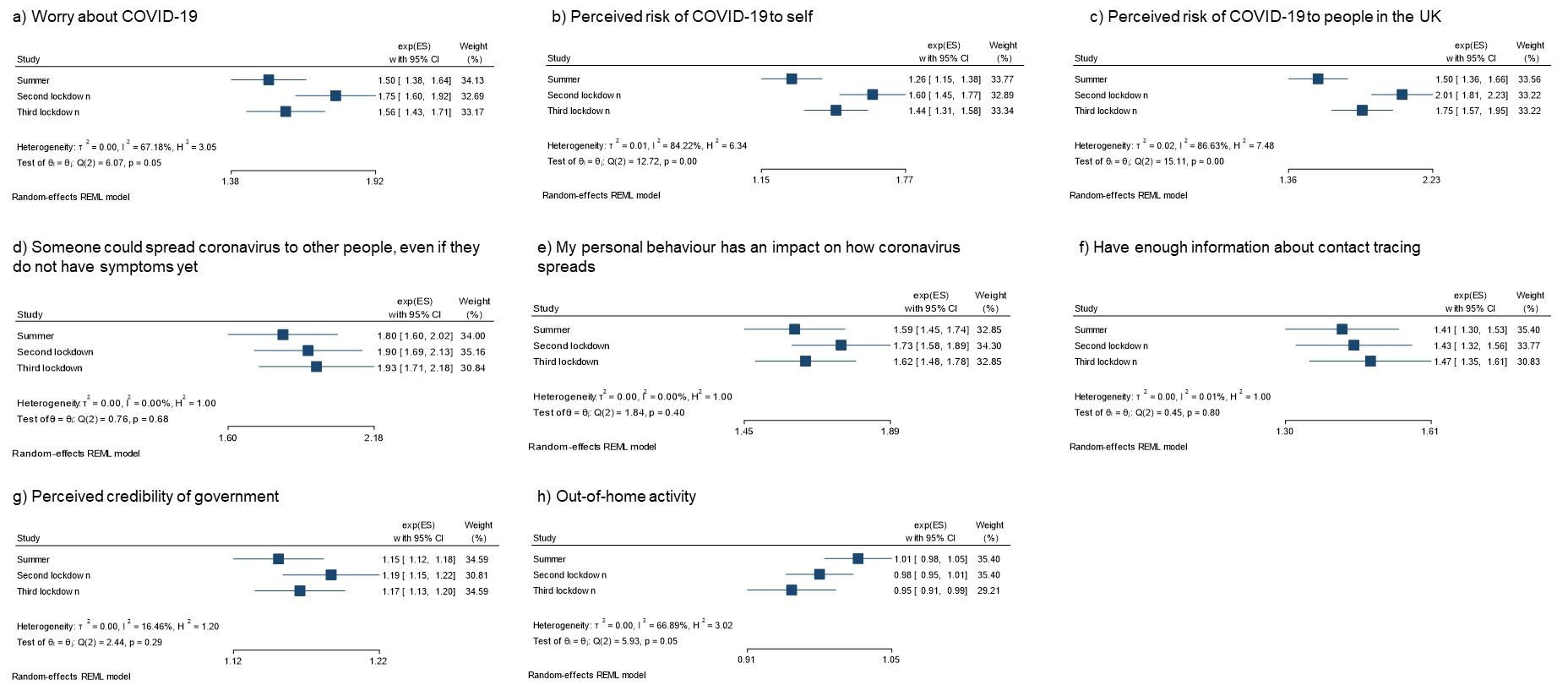


# Supplementary materials. Associations between personal and clinical characteristics and outcomes in fully adjusted regression models.

## Intention to self-isolate

Table 1. Personal and clinical characteristics associated with intention to self-isolate in fully adjusted regression models, for each timepoint in the pandemic. Bolding indicates findings significant at p<0.007.

|  |  | First lockdown ^a^ | | Summer, fewest restrictions ^b^ | | Second lockdown ^c^ | | Third lockdown ^d^ | |
| --- | --- | --- | --- | --- | --- | --- | --- | --- | --- |
| Attribute | Level | aOR for intending to self-isolate (95% CI) † | *p* | aOR for intending to self-isolate (95% CI) ‡ | *p* | aOR for intending to self-isolate (95% CI) ‡ | *p* | aOR for intending to self-isolate (95% CI) ‡ | *p* |
| Survey wave | Wave 1 in timepoint | Ref | - | Ref | - | Ref | - | Ref | - |
|  | Wave 2 in timepoint | 0.96 (0.78 to 1.19) | .73 | 1.28 (1.06 to 1.54) | .01 | 1.02 (0.86 to 1.20) | .84 | 1.02 (0.86 to 1.21) | .83 |
| Region | East Midlands | Ref | - | Ref | - | Ref | - | Ref | - |
|  | East of England | 0.76 (0.46 to 1.24) | .27 | 0.72 (0.46 to 1.12) | .14 | 1.06 (0.74 to 1.53) | .74 | 1.04 (0.71 to 1.52) | .84 |
|  | London | 0.68 (0.43 to 1.10) | .12 | 0.68 (0.45 to 1.05) | .08 | 0.86 (0.60 to 1.23) | .40 | 0.68 (0.46 to 0.99) | .05 |
|  | North East | 0.70 (0.39 to 1.25) | .22 | 0.87 (0.50 to 1.51) | .61 | 0.79 (0.52 to 1.21) | .28 | 0.90 (0.56 to 1.44) | .67 |
|  | North West | 0.86 (0.53 to 1.40) | .55 | 0.76 (0.49 to 1.18) | .22 | 0.97 (0.68 to 1.39) | .87 | 0.95 (0.65 to 1.38) | .79 |
|  | South East | 0.61 (0.39 to 0.97) | .04 | 0.83 (0.54 to 1.26) | .38 | 1.47 (1.03 to 2.10) | .03 | 1.27 (0.88 to 1.83) | .20 |
|  | South West | 0.68 (0.42 to 1.11) | .13 | 0.73 (0.46 to 1.15) | .18 | 0.94 (0.64 to 1.37) | .74 | 0.82 (0.56 to 1.20) | .30 |
|  | West Midlands | 0.67 (0.42 to 1.09) | .11 | 0.77 (0.49 to 1.20) | .25 | 0.76 (0.52 to 1.11) | .16 | 0.79 (0.54 to 1.17) | .24 |
|  | Yorkshire and The Humber | 1.22 (0.72 to 2.05) | .46 | 0.77 (0.49 to 1.21) | .26 | 0.85 (0.58 to 1.24) | .39 | 1.09 (0.74 to 1.61) | .66 |
|  | Overall | χ^2^(8)=13.6 | .09 | χ^2^(8)=4.0 | .85 | χ^2^(8)=19.5 | .01 | χ^2^(8)=18.5 | .02 |
| Gender | Male | Ref | - | Ref | - | Ref | - | Ref | - |
|  | Female | **1.47 (1.18 to 1.82)** | **.001** | 1.31 (1.08 to 1.58) | .007 | 1.24 (1.04 to 1.47) | .02 | 1.20 (1.00 to 1.43) | .04 |
| Age (per decade) | Raw age | 1.00 (0.92 to 1.09) | .98 | 1.01 (0.93 to 1.08) | .88 | 0.97 (0.90 to 1.04) | .33 | 0.99 (0.92 to 1.06) | .68 |
| Age: quadratic (age-mean)^2^ | - | 1.0001 (0.9997 to 1.0005) | .59 | 1.0003 (0.9999 to 1.0006) | .15 | 1.0000 (0.9997 to 1.0003) | 1.00 | **0.9995 (0.9992 to 0.9998)** | **.001** |
| Dependent child in household | None | Ref | - | Ref | - | Ref | - | Ref | - |
|  | Child present | 0.73 (0.56 to 0.94) | .02 | 0.97 (0.78 to 1.22) | .81 | 0.90 (0.73 to 1.11) | .34 | **0.73 (0.59 to 0.91)** | **.004** |
| Clinically vulnerable to COVID-19 | No | Ref | - | Ref | - | Ref | - | Ref | - |
|  | Yes | 1.12 (0.84 to 1.51) | .44 | 1.26 (0.96 to 1.66) | .09 | **1.40 (1.10 to 1.77)** | **.006** | 1.30 (1.02 to 1.67) | .03 |
| Household member has chronic illness | No | Ref | - | Ref | - | Ref | - | Ref | - |
|  | Yes | 1.19 (0.88 to 1.60) | .26 | 1.03 (0.80 to 1.34) | .80 | 1.39 (1.09 to 1.77) | .008 | 1.20 (0.94 to 1.54) | .14 |
| Employment status | Not working | Ref | - | Ref | - | Ref | - | Ref | - |
|  | Working | 0.99 (0.76 to 1.29) | .93 | 0.83 (0.66 to 1.04) | .11 | 0.80 (0.65 to 0.99) | .04 | 0.90 (0.72 to 1.12) | .35 |
| Socio-economic grade‡ | ABC1 | Ref | - | Ref | - | Ref | - | Ref | - |
|  | C2DE | 0.96 (0.75 to 1.22) | .72 | 0.76 (0.62 to 0.95) | .01 | 0.81 (0.67 to 0.98) | .03 | 1.00 (0.81 to 1.23) | .99 |
| Index of multiple deprivation | 1^st^ (least) to 4^th^ quartile (most deprived) | 0.96 (0.87 to 1.06) | .44 | 0.98 (0.89 to 1.07) | .65 | 0.94 (0.87 to 1.02) | .16 | 0.95 (0.87 to 1.03) | .20 |
| Highest educational or professional qualification | Less than degree | Ref | - | Ref | - | Ref | - | Ref | - |
|  | Degree or higher | 0.98 (0.78 to 1.24) | .88 | 1.04 (0.84 to 1.28) | 0.72 | 1.07 (0.88 to 1.29) | .50 | 0.89 (0.74 to 1.08) | .25 |
| Ethnicity | White British | Ref | - | Ref | - | Ref | - | Ref | - |
|  | White other | 0.71 (0.47 to 1.07) | .10 | 0.85 (0.59 to 1.24) | .41 | 0.83 (0.58 to 1.18) | .29 | 1.04 (0.72 to 1.50) | .84 |
|  | Black and minority ethnicity | 1.14 (0.77 to 1.70) | .51 | 0.96 (0.69 to 1.35) | .83 | 0.86 (0.63 to 1.17) | .34 | 1.28 (0.92 to 1.78) | .14 |
|  | Overall | χ^2^(2)=3.5 | .17 | χ^2^(2)=0.7 | .71 | χ^2^(2)=1.7 | .42 | χ^2^(2)=2.2 | .34 |
| Living alone | Not living alone | Ref | - | Ref | - | Ref | - | Ref | - |
|  | Living alone | 0.96 (0.68 to 1.36) | .83 | 0.92 (0.68 to 1.25) | .60 | 1.38 (1.04 to 1.85) | .03 | 0.72 (0.54 to 0.96) | .03 |
| Marital status | Not partnered | Ref | - | Ref | - | Ref | - | Ref | - |
|  | Partnered | 1.11 (0.84 to 1.45) | .46 | 1.24 (0.98 to 1.57) | .07 | 1.23 (0.98 to 1.54) | .08 | 0.79 (0.63 to 1.00) | .05 |
| Ever had COVID-19 | Think not | Ref | - | Ref | - | Ref | - | Ref | - |
|  | Think yes | 0.79 (0.56 to 1.12) | .18 | 0.79 (0.60 to 1.04) | .09 | 0.80 (0.62 to 1.02) | .08 | 0.93 (0.73 to 1.19) | .57 |
| Financial hardship | Range 3 (least) to 15 (most) | **0.87 (0.83 to 0.91)** | **<.001** | **0.91 (0.88 to 0.94)** | **<.001** | 0.96 (0.93 to 0.99) | .02 | 0.96 (0.93 to 1.00) | .03 |

† All variables entered into regression model together (personal and clinical characteristics), excluding perceived adequacy of information about self-isolation.

‡ All variables entered into regression model together (personal and clinical characteristics), including perceived adequacy of information about self-isolation.

a. Model based on 2694 valid cases (83.5% valid responses).

b. Model based on 2645 valid cases (81.6% valid responses).

c. Model based on 2742 valid cases (83.2% valid responses).

d. Model based on 2704 valid cases (84.1% valid responses).

## Intention to request a test

Table 2. Personal and clinical characteristics associated with intention to request a test in fully adjusted regression models, for each timepoint in the pandemic. Bolding indicates findings significant at p<0.007.

|  |  | Summer, fewest restrictions ^a^ | | Second lockdown ^b^ | | Third lockdown ^c^ | |
| --- | --- | --- | --- | --- | --- | --- | --- |
| Attribute | Level | aOR for intending to request a test (95% CI) † | *p* | aOR for intending to request a test (95% CI) † | *p* | aOR for intending to request a test (95% CI) † | *p* |
| Survey wave | Wave 1 in timepoint | Ref | - | Ref | - | Ref | - |
|  | Wave 2 in timepoint | 1.15 (0.98 to 1.35) | .10 | 1.00 (0.85 to 1.18) | .97 | 1.08 (0.92 to 1.28) | .36 |
| Region | East Midlands | Ref | - | Ref | - | Ref | - |
|  | East of England | 1.22 (0.85 to 1.75) | .27 | 0.95 (0.67 to 1.36) | .80 | 1.08 (0.74 to 1.56) | .70 |
|  | London | 0.94 (0.66 to 1.33) | .72 | 0.67 (0.47 to 0.95) | .02 | 0.82 (0.57 to 1.20) | .31 |
|  | North East | 1.51 (0.96 to 2.36) | .07 | 0.85 (0.56 to 1.30) | .46 | 0.79 (0.50 to 1.25) | .32 |
|  | North West | 1.07 (0.76 to 1.52) | .69 | 0.99 (0.70 to 1.42) | .97 | 0.94 (0.65 to 1.36) | .74 |
|  | South East | 1.30 (0.92 to 1.82) | .13 | 1.09 (0.77 to 1.53) | .62 | 1.04 (0.73 to 1.47) | .84 |
|  | South West | 0.98 (0.68 to 1.42) | .92 | 0.85 (0.59 to 1.23) | .39 | 1.15 (0.78 to 1.68) | .48 |
|  | West Midlands | 0.87 (0.61 to 1.25) | .46 | 0.98 (0.67 to 1.42) | .91 | 0.91 (0.62 to 1.33) | .62 |
|  | Yorkshire and The Humber | 1.11 (0.77 to 1.59) | .58 | 0.92 (0.64 to 1.34) | .67 | 0.79 (0.54 to 1.14) | .21 |
|  | Overall | χ^2^(8)=12.6 | .13 | χ^2^(8)=12.0 | .15 | χ^2^(8)=8.3 | .40 |
| Gender | Male | Ref | - | Ref | - | Ref | - |
|  | Female | **1.50 (1.27 to 1.77)** | **<.001** | **1.40 (1.18 to 1.65)** | **<.001** | **1.55 (1.31 to 1.84)** | **<.001** |
| Age (per decade) | Raw age | 1.00 (0.94 to 1.06) | .95 | 0.92 (0.86 to 0.99) | .02 | 0.93 (0.87 to 1.00) | .04 |
| Age: quadratic (age-mean)^2^ | - | 1.0002 (0.9999 to 1.0005) | .30 | 0.9999 (0.9996 to 1.0003) | .74 | 0.9998 (0.9995 to 1.0001) | .12 |
| Dependent child in household | None | Ref | - | Ref | - | Ref | - |
|  | Child present | 0.99 (0.81 to 1.21) | .92 | 1.06 (0.87 to 1.31) | .55 | 0.98 (0.80 to 1.21) | .87 |
| Clinically vulnerable to COVID-19 | No | Ref | - | Ref | - | Ref | - |
|  | Yes | 1.22 (0.98 to 1.53) | .08 | 0.97 (0.78 to 1.21) | .78 | 1.19 (0.95 to 1.50) | .14 |
| Household member has chronic illness | No | Ref | - | Ref | - | Ref | - |
|  | Yes | 0.98 (0.79 to 1.22) | .86 | 1.08 (0.85 to 1.36) | .54 | 1.23 (0.97 to 1.56) | .09 |
| Employment status | Not working | Ref | - | Ref | - | Ref | - |
|  | Working | 0.95 (0.79 to 1.16) | .64 | 0.97 (0.79 to 1.19) | .77 | 1.18 (0.95 to 1.45) | .13 |
| Socio-economic grade‡ | ABC1 | Ref | - | Ref | - | Ref | - |
|  | C2DE | 0.87 (0.72 to 1.05) | .15 | 0.88 (0.72 to 1.06) | .18 | 0.89 (0.73 to 1.09) | .25 |
| Index of multiple deprivation | 1^st^ (least) to 4^th^ quartile (most deprived) | 0.95 (0.88 to 1.02) | .16 | 0.90 (0.83 to 0.97) | .008 | 0.92 (0.85 to 1.00) | .05 |
| Highest educational or professional qualification | Less than degree | Ref | - | Ref | - | Ref | - |
|  | Degree or higher | **1.35 (1.13 to 1.62)** | **.001** | 0.91 (0.76 to 1.10) | .34 | 0.84 (0.70 to 1.02) | .07 |
| Ethnicity | White British | Ref | - | Ref | - | Ref | - |
|  | White other | 0.68 (0.48 to 0.95) | .03 | 0.68 (0.48 to 0.96) | .03 | 0.83 (0.58 to 1.19) | .32 |
|  | Black and minority ethnicity | 0.70 (0.52 to 0.96) | .03 | 0.76 (0.56 to 1.04) | .09 | 0.68 (0.50 to 0.94) | .02 |
|  | Overall | χ^2^(2)=8.5 | .01 | χ^2^(2)=6.7 | .04 | χ^2^(2)=6.0 | .05 |
| Living alone | Not living alone | Ref | - | Ref | - | Ref | - |
|  | Living alone | 1.13 (0.86 to 1.48) | .40 | 0.93 (0.70 to 1.23) | .59 | 0.81 (0.61 to 1.08) | .15 |
| Marital status | Not partnered | Ref | - | Ref | - | Ref | - |
|  | Partnered | 1.14 (0.93 to 1.40) | .22 | 1.10 (0.88 to 1.38) | .40 | 1.01 (0.81 to 1.27) | .91 |
| Ever had COVID-19 | Think not | Ref | - | Ref | - | Ref | - |
|  | Think yes | 0.88 (0.69 to 1.14) | .34 | 0.87 (0.68 to 1.12) | .28 | 0.98 (0.76 to 1.25) | .86 |
| Financial hardship | Range 3 (least) to 15 (most) | **0.94 (0.91 to 0.97)** | **<.001** | **0.94 (0.91 to 0.97)** | **<.001** | **0.93 (0.90 to 0.96)** | **<.001** |

† All variables entered into regression model together (personal and clinical characteristics, and other psychological factors).

a. Model based on 2646 valid cases (81.7% valid responses).

b. Model based on 2732 valid cases (82.9% valid responses).

c. Model based on 2702 valid cases (84.0% valid responses).

## Intention to share details of close contacts

Table 3. Personal and clinical characteristics associated with intention to share details of close contacts in fully adjusted regression models, for each timepoint in the pandemic. Bolding indicates findings significant at p<0.007.

|  |  | Summer, fewest restrictions ^a^ | | Second lockdown ^b^ | | Third lockdown ^c^ | |
| --- | --- | --- | --- | --- | --- | --- | --- |
| Attribute | Level | aOR for intending to share details (95% CI) † | *p* | aOR for intending to share details (95% CI) † | *p* | aOR for intending to share details (95% CI) † | *p* |
| Survey wave | Wave 1 in timepoint | Ref | - | Ref | - | Ref | - |
|  | Wave 2 in timepoint | 1.15 (0.94 to 1.42) | .18 | 1.29 (1.04 to 1.60) | .02 | 1.2 (0.97 to 1.49) | .10 |
| Region | East Midlands | Ref | - | Ref | - | Ref | - |
|  | East of England | 1.09 (0.67 to 1.76) | .74 | 0.86 (0.55 to 1.36) | .53 | 0.89 (0.55 to 1.45) | .65 |
|  | London | 0.85 (0.54 to 1.35) | .50 | 0.97 (0.62 to 1.53) | .90 | 0.78 (0.48 to 1.27) | .32 |
|  | North East | 1.52 (0.80 to 2.89) | .20 | 1.32 (0.75 to 2.34) | .34 | 1.02 (0.56 to 1.87) | .95 |
|  | North West | 1.05 (0.66 to 1.68) | .82 | 1.36 (0.85 to 2.18) | .20 | 1.19 (0.73 to 1.94) | .49 |
|  | South East | 0.88 (0.56 to 1.38) | .58 | 1.57 (0.99 to 2.49) | .05 | 1.09 (0.68 to 1.74) | .73 |
|  | South West | 1.17 (0.70 to 1.97) | .54 | 1.12 (0.68 to 1.83) | .65 | 1.13 (0.68 to 1.90) | .63 |
|  | West Midlands | 0.73 (0.45 to 1.16) | .18 | 0.74 (0.46 to 1.18) | .21 | 0.85 (0.51 to 1.41) | .53 |
|  | Yorkshire and The Humber | 0.89 (0.55 to 1.43) | .63 | 1.15 (0.70 to 1.89) | .57 | 0.78 (0.48 to 1.26) | .31 |
|  | Overall | χ^2^(8)=9.8 | .28 | χ^2^(8)=16.9 | .03 | χ^2^(8)=7.7 | .46 |
| Gender | Male | Ref | - | Ref | - | Ref | - |
|  | Female | **1.43 (1.16 to 1.77)** | **.001** | 1.17 (0.94 to 1.47) | .16 | 0.97 (0.78 to 1.21) | .78 |
| Age (per decade) | Raw age | **1.23 (1.13 to 1.34)** | **<.001** | **1.16 (1.06 to 1.28)** | **.002** | 1.08 (0.99 to 1.18) | .09 |
| Age: quadratic (age-mean)^2^ | - | **1.0007 (1.0003 to 1.0012)** | **.001** | **1.001 (1.0006 to 1.0015)** | **<.001** | **1.0006 (1.0002 to 1.0010)** | **.003** |
| Dependent child in household | None | Ref | - | Ref | - | Ref | - |
|  | Child present | 0.98 (0.77 to 1.25) | .86 | 1.44 (1.10 to 1.88) | .008 | 1.10 (0.84 to 1.42) | .49 |
| Clinically vulnerable to COVID-19 | No | Ref | - | Ref | - | Ref | - |
|  | Yes | 1.34 (0.98 to 1.83) | .07 | 1.22 (0.89 to 1.69) | .22 | 1.29 (0.94 to 1.78) | .12 |
| Household member has chronic illness | No | Ref | - | Ref | - | Ref | - |
|  | Yes | 1.07 (0.80 to 1.43) | .66 | 1.09 (0.80 to 1.49) | .59 | 1.57 (1.13 to 2.18) | .008 |
| Employment status | Not working | Ref | - | Ref | - | Ref | - |
|  | Working | 0.86 (0.67 to 1.11) | .24 | 1.20 (0.92 to 1.57) | .17 | 1.43 (1.10 to 1.86) | .008 |
| Socio-economic grade‡ | ABC1 | Ref | - | Ref | - | Ref | - |
|  | C2DE | 0.88 (0.69 to 1.12) | .29 | 0.79 (0.62 to 1.01) | .06 | 1.05 (0.82 to 1.36) | .69 |
| Index of multiple deprivation | 1^st^ (least) to 4^th^ quartile (most deprived) | 0.96 (0.87 to 1.06) | .44 | 1.01 (0.91 to 1.13) | .82 | 0.89 (0.80 to 0.99) | .03 |
| Highest educational or professional qualification | Less than degree | Ref | - | Ref | - | Ref | - |
|  | Degree or higher | **1.47 (1.17 to 1.86)** | **.001** | 1.03 (0.81 to 1.32) | .79 | 1.07 (0.84 to 1.37) | .59 |
| Ethnicity | White British | Ref | - | Ref | - | Ref | - |
|  | White other | 0.69 (0.47 to 1.02) | .06 | 0.97 (0.62 to 1.51) | .90 | **2.10 (1.25 to 3.53)** | **.005** |
|  | Black and minority ethnicity | 0.75 (0.52 to 1.08) | .12 | 0.60 (0.42 to 0.88) | .008 | 0.85 (0.58 to 1.24) | .40 |
|  | Overall | χ^2^(2)=4.9 | .08 | χ^2^(2)=7.1 | .03 | χ^2^(2)=9.5 | .009 |
| Living alone | Not living alone | Ref | - | Ref | - | Ref | - |
|  | Living alone | 0.95 (0.68 to 1.33) | .75 | 1.00 (0.69 to 1.44) | 1.00 | 1.18 (0.83 to 1.68) | .36 |
| Marital status | Not partnered | Ref | - | Ref | - | Ref | - |
|  | Partnered | 1.34 (1.04 to 1.73) | .02 | 0.97 (0.72 to 1.30) | .84 | 1.15 (0.87 to 1.51) | .33 |
| Ever had COVID-19 | Think not | Ref | - | Ref | - | Ref | - |
|  | Think yes | 0.97 (0.72 to 1.31) | .86 | 1.04 (0.75 to 1.43) | .81 | 0.96 (0.71 to 1.30) | .80 |
| Financial hardship | Range 3 (least) to 15 (most) | 1.02 (0.97 to 1.06) | .48 | 0.97 (0.93 to 1.01) | .13 | 0.96 (0.92 to 1.01) | .09 |

† All variables entered into regression model together (personal and clinical characteristics, and other psychological factors).

a. Model based on 2640 valid cases (81.5% valid responses).

b. Model based on 2733 valid cases (82.9% valid responses).

c. Model based on 2695 valid cases (83.8% valid responses).
